# Supplementary material for: Conserved heat shock factors HvHSFA2 and HvHSFA3 control barley heat stress memory through diverged mechanisms
Source: Nat Commun. 2025 Nov 23;16:10411. doi: 10.1038/s41467-025-66651-6 (PMC12644563; doi:10.1038/s41467-025-66651-6)
Supplement: Supplementary file 1 — Supplementary Information [file 41467_2025_66651_MOESM1_ESM.pdf]

Supplementary Information

Conserved heat shock factors HvHSFA2 and HvHSFA3 control barley heat stress memory through diverged mechanisms

Loris Pratx[1], Yuri Dakhiya[1], Ruqayyah Nissen[1], Preethi Purushotham[2], Iris Hoffie[2], Jochen Kumlehn[2], Christian Kappel[1], Isabel Bäurle[1*]

Supplementary Figures

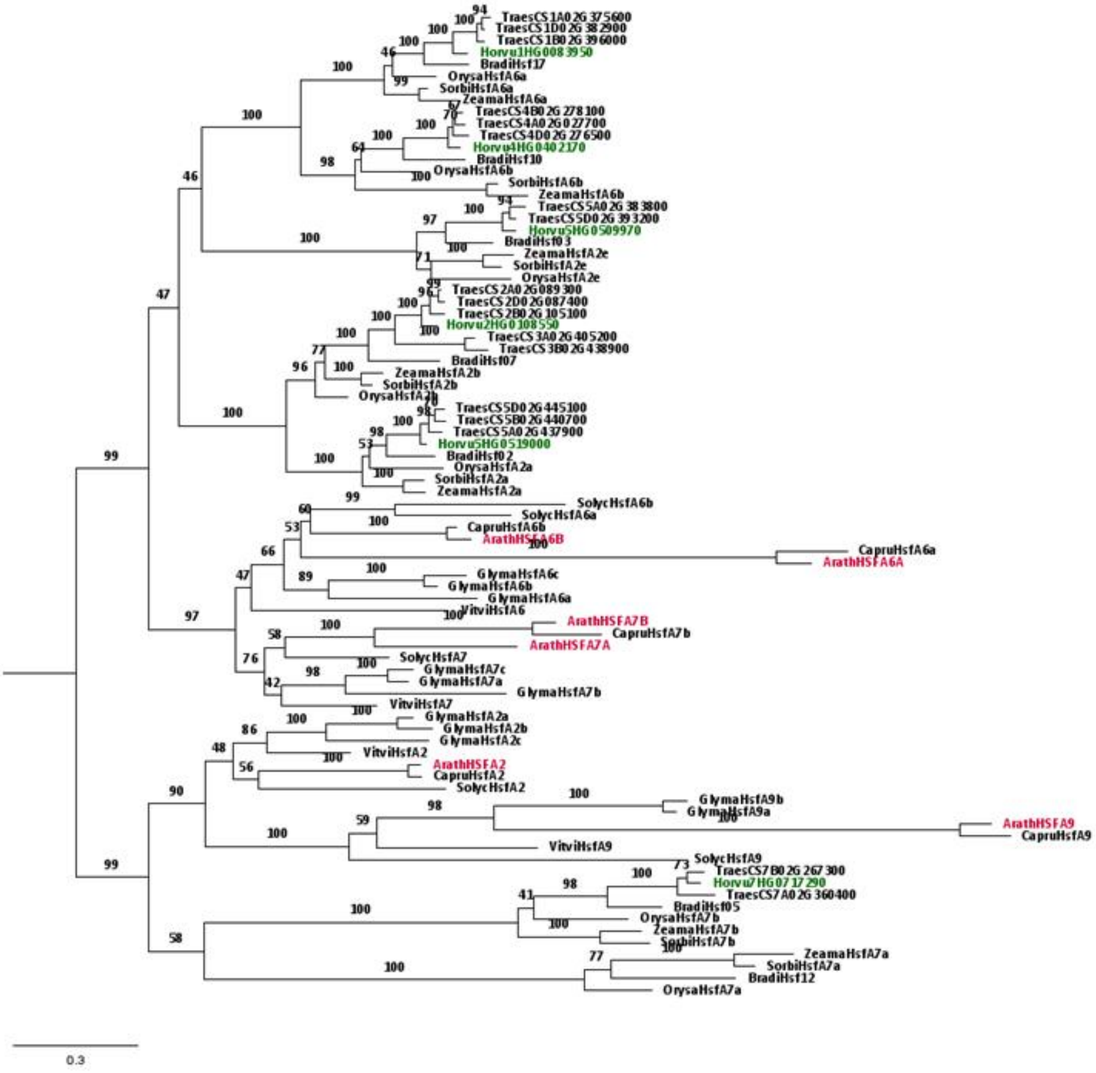

Supplementary Fig. 1: Phylogenetic tree of HSFA2/6/7/9

From the phylogenetic tree of all HSFAs between 10 species (*A. thaliana*, *Brachypodium distachyon*, *Capsella rubella*, *H. vulgare*, *Oryza sativa*, *Solanum lycopersicum*, *Sorghum bicolor*, *Triticum aestivum*, *Vitis vinifera* and *Zea mays*; Fig. 2a), we extracted the branch containing HSFA2/6/7/9 to build a new phylogeny. From this, we identified dicot HSFA2 and HSFA9 as orthologous with HSFA7a and HSFA7b in monocots and 7HG0717290 as the closest barley ortholog for HSFA2.

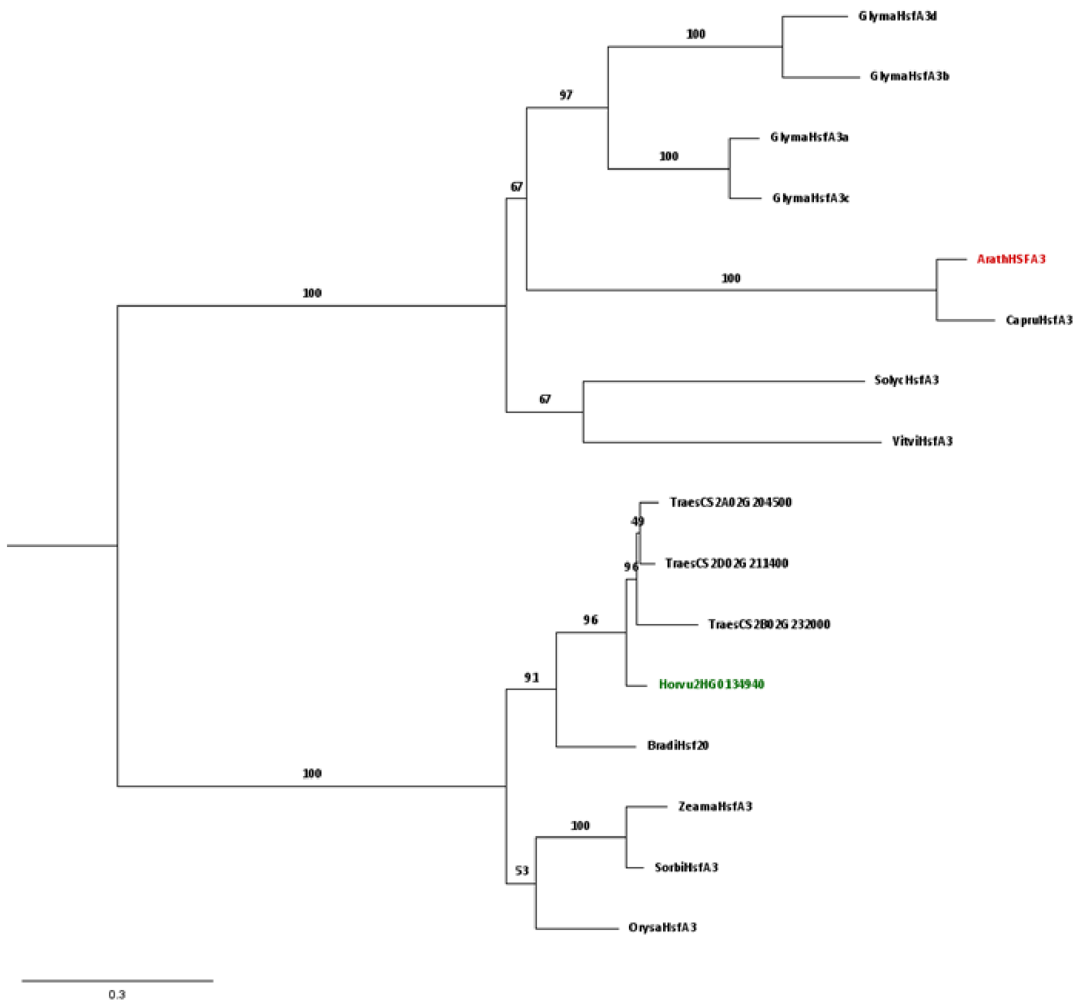

Supplementary Fig. 2: Phylogenetic tree of HSFA3

From the phylogenetic tree of all HSFAs between 10 species (*A. thaliana*, *Brachypodium distachyon*, *Capsella rubella*, *H. vulgare*, *Oryza sativa*, *Solanum lycopersicum*, *Sorghum bicolor*, *Triticum aestivum*, *Vitis vinifera* and *Zea mays*; Fig. 2a), we extracted the branch containing HSFA3 to build a new phylogeny. From this, we identified 2HG0134940 as the ortholog of HSFA3 in barley.

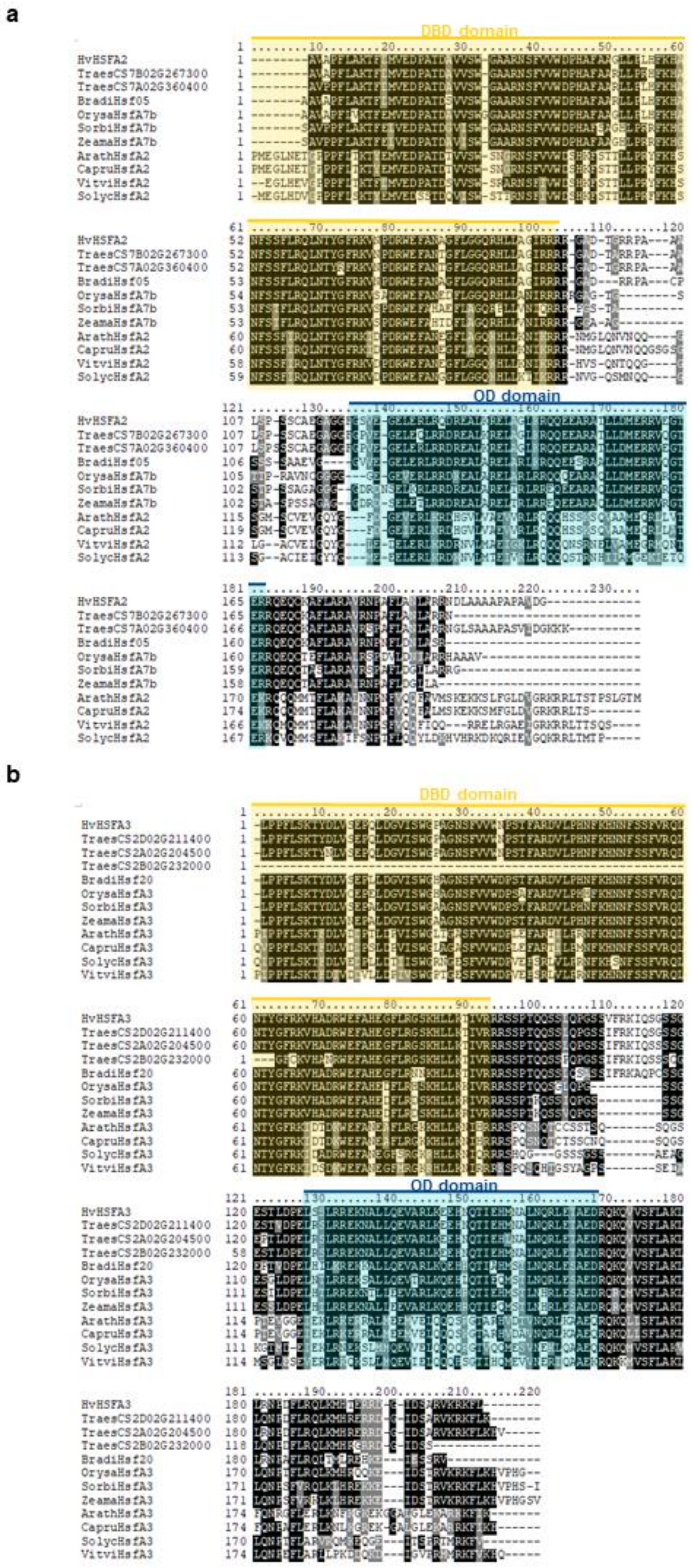

Supplementary Fig. 3: HSF domain alignment of HSFA2 (a) and HSFA3 (b) orthologs across 10 species

Multiple sequence alignment of the HSF domain of HSFA2 (a) and HSFA3 (b) from 10 species (*A. thaliana*, *Brachypodium distachyon*, *Capsella rubella*, *H. vulgare*, *Oryza sativa*, *Solanum lycopersicum*, *Sorghum bicolor*, *Triticum aestivum*, *Vitis vinifera* and *Zea mays*), cf. Fig. 2a,

Supplementary Figs. S1, 2. The DNA-binding domain (DBD) and oligomerization domain (OD) are highlighted in yellow and blue, respectively.

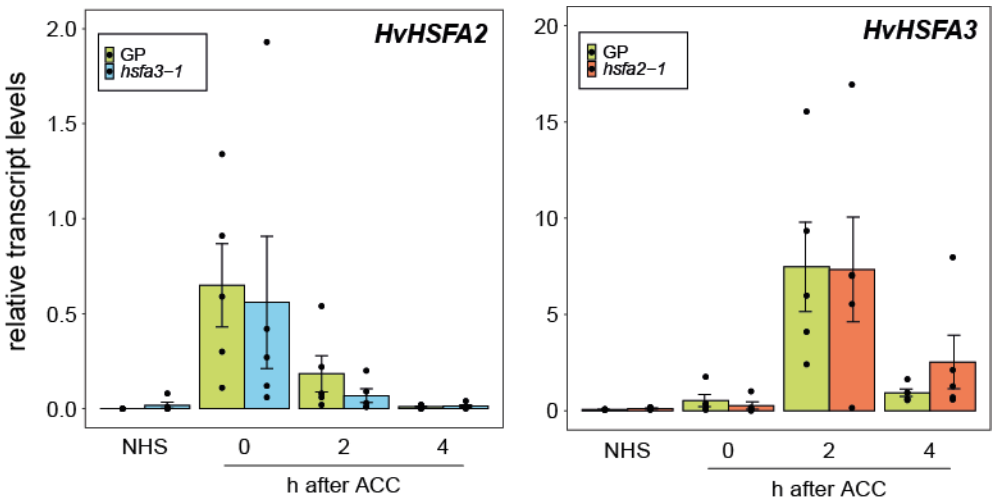

Supplementary Fig. 4: HSFA2 and HSFA3 do not regulate each other's expression
Transcript levels of *HSFA2* in wild type (GP) and *hsfa3-1* without, or 0 to 4 h after ACC. Transcript levels of *HSFA3* in GP and *hsfa2-1* without, or 0 to 4 h after ACC. No significant differences (Welch two-sided t-test) were observed at any time point between GP and mutant, suggesting that *HSFA2* and *HSFA3* are not required for each other's expression. Data are mean ± SEM of five independent replicates.

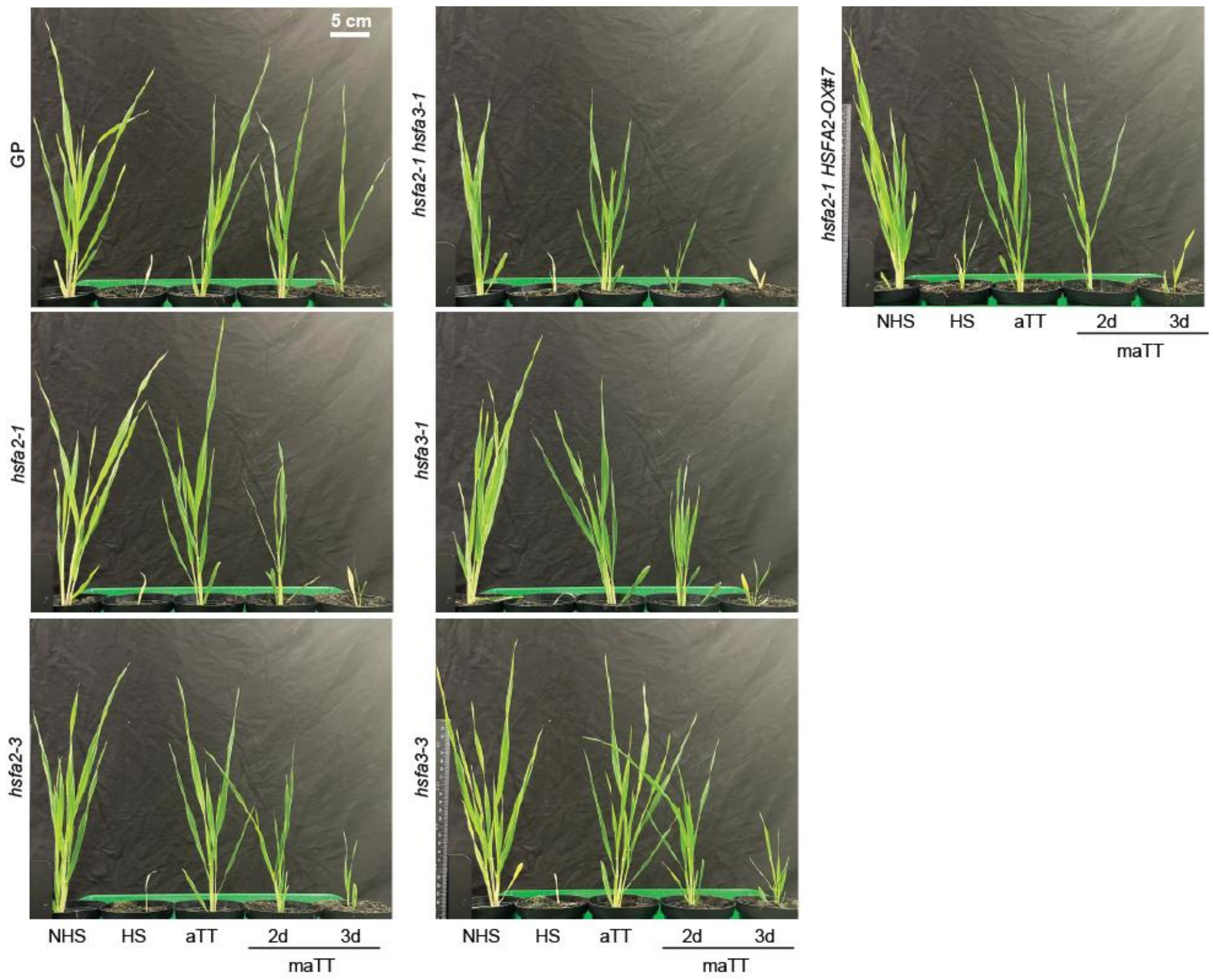

Supplementary Fig. 5: Comparison of growth phenotypes 21 d after HS in different genotypes

Representative photographs of wild type barley (GP) and the indicated mutants after different HS treatments. Representative plants are shown for each genotype and treatment condition. Priming and HS treatments were performed as indicated in Fig. 1a. Pictures were taken 21 d after HS. Plants were grown together with those in Fig. 7e and GP is the same picture as shown in Fig. 7e.

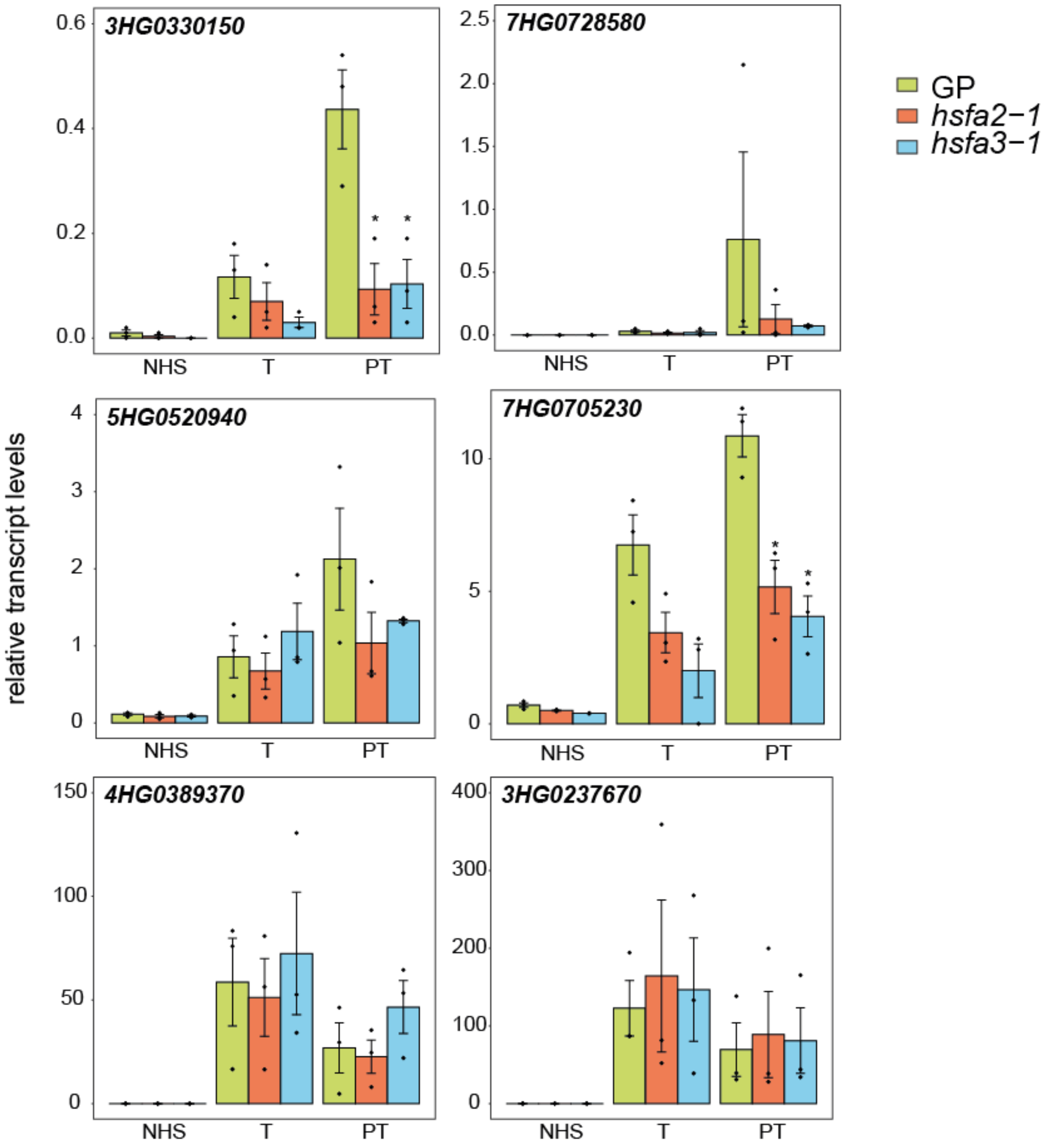

Supplementary Fig. 6: qRT-PCR analysis of type II HS memory genes

Transcript levels in 4 type II memory genes were analyzed by qRT-PCR; a MYB transcription factor (3HG0330150), two ATP-dependent zinc metalloproteases (7HG0728580, 5HG0520940), and an E3 ubiquitin-protein ligase (7HG0705230). Two non-memory HS-inducible HSP genes (4HG0389370 and H3HG0237670) were also analyzed. The four HS memory genes showed a stronger induction after a second ACC treatment (P+T) than after a single one (T, ACC). This stronger response was attenuated in *hsfa2-1* and *hsfa3-1* mutants. Data are mean relative transcript levels ± SEM of three independent replicates. Asterisk indicates significant differences compared with GP wild type (*, $p < 0.05$; unpaired two-sided t-test).

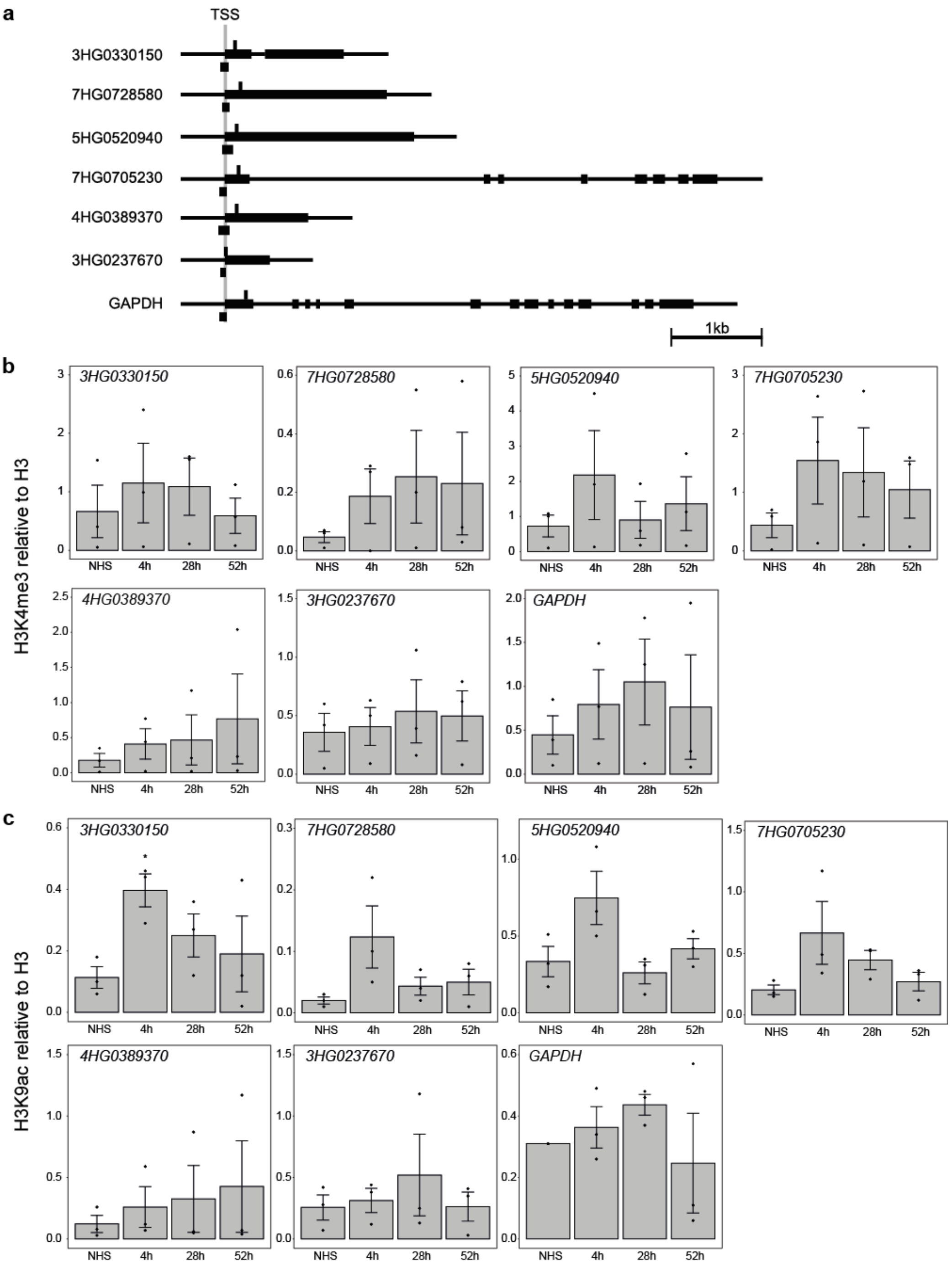

Supplementary Fig. 7: Chromatin analysis of memory and HS-responsive genes
Four validated type II memory genes (*3HG0330150*, *7HG0728580*, *5HG0520940* and *7HG0705230,* (**a**)) were analyzed for the deposition and maintenance of H3K4me3 (**b**) and H3K9ac (**c**) marks after ACC treatment. Two non-memory *HSP* (*4HG0389370* and *3HG0237670*) and *GAPDH* were also included. (**a**) Localization of amplicons used for ChIP-qPCR assays. All amplicons are localized at the transcription start site (TSS). Vertical bars indicate the translation initiation site. (**b**) None of the tested genes exhibited enrichment in H3K4me3 after ACC. (**c**) All memory genes showed enrichment in H3K9ac immediately after ACC, one of which passing the significance threshold (*3HG0330150*) compared with NHS (*, $p < 0.05$; unpaired two-sided t-test). Data are mean ± SEM of three independent biological replicates.

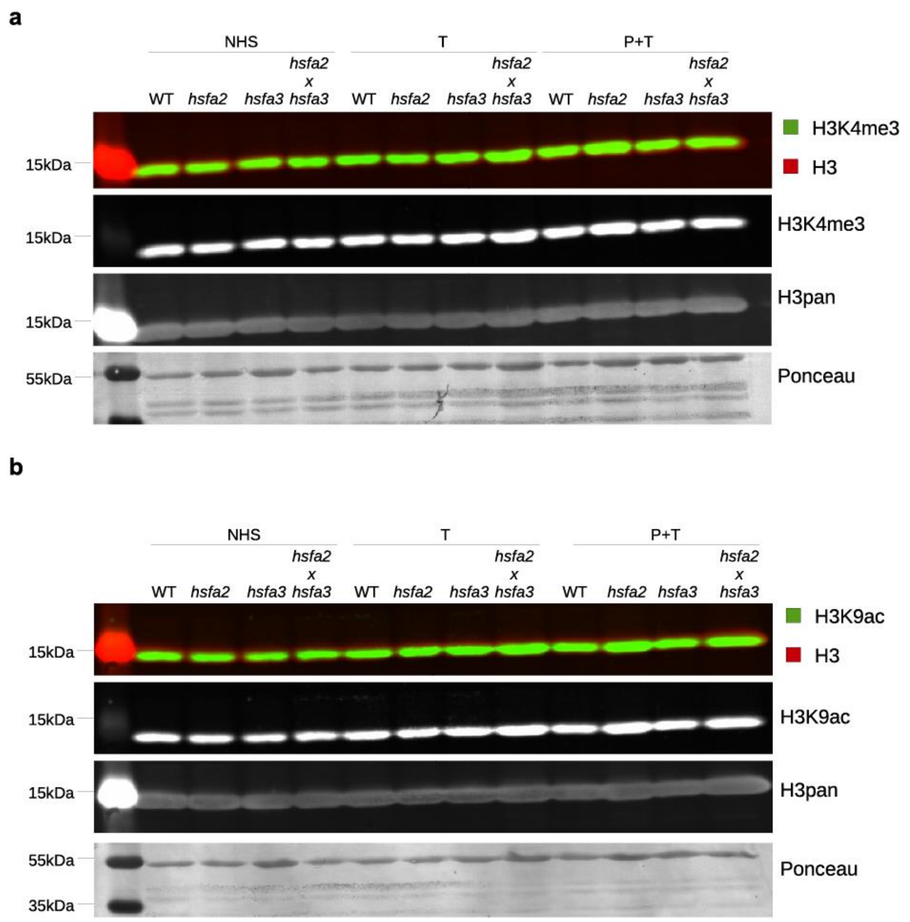

Supplementary Fig. 8: Global levels of H3K4me3 (a) or H3K9ac (b) are unaffected by heat treatment or loss of *HSFA2/HSFA3*

The global accumulation of H3K4me3 (**a**) or H3K9ac (**b**) was assessed by immunoblotting after single (T) or recurring (PT) ACC treatment, in wild type, *hsfa2-1, hsfa3-1* and *hsfa2-1 hsfa3-1* double mutant. The top panel shows the overlay of anti-H3(modified) and anti-H3pan, which are shown individually in bottom panels. No difference was visible between genotypes or treatments. H3pan antibodies and Ponceau staining were used as loading controls. The experiment was repeated with similar results.

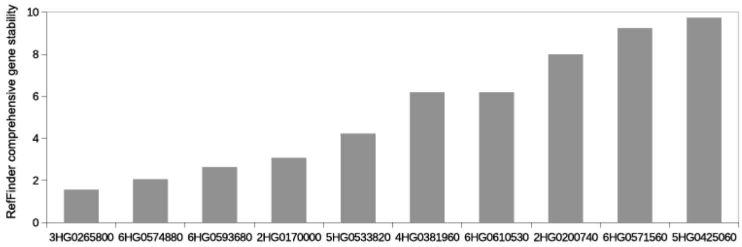

Supplementary Fig. 9: Reference gene stability analysis
The stability of 10 genes among non-treated and treated (N, T and PT), and among wild type and mutants (*hsfa2-1, hsfa3-1*) was tested by qRT-PCR. Gene stability was calculated using RefFinder. *3HG0265800*, *6HG0574880* and *6HG0593680* were identified as the most stable genes under our conditions and used together as reference genes in all qRT-PCR assays.
