# Supplementary material for: Conserved heat shock factors HvHSFA2 and HvHSFA3 control barley heat stress memory through diverged mechanisms
Source: Nat Commun. 2025 Nov 23;16:10411. doi: 10.1038/s41467-025-66651-6 (PMC12644563; doi:10.1038/s41467-025-66651-6)
Supplement: Supplementary file 2 — Description of Additional Supplementary Files [file 41467_2025_66651_MOESM2_ESM.pdf]

Description of Additional Supplementary Files

Supplementary Data 1: HSFA sequences

Supplementary Data 2: RNA-seq gene list

Supplementary Data 3: Oligonucleotide sequences Source data file
